# Intelligence

**Published:** 1808-12

**Authors:** 


### INTELLIGENCE.

At the Anniversary Meeting of the Colchester Medical Society, holden this day, August 23, 1808, the following Resolutions were entered on their Journals, in consequence of a motion made at their last Meeting, June 21st, by Dr. NEWELL, requesting the Members of the Society, at their next Meeting, to Report their Observations on the Security of Vaccination as a Protection from Small Pox, and the consequent Effects of it upon the Constitution.

"Resolved, That the Members of this Society, from a steady, candid and impartial attention to the effects of Vaccination, during a period of eight years, in which time several thousand persons have been vaccinated by them, think it their duty to give this public testimony of its perfect safety and security as a preventive of Small-pox; and further, that under the fullest conviction of the practice, they have not only recommended it to their patients, but introduced and practised it in their own families, amongst their nearest and dearest relatives. That they have at different times exposed many of their patients, long afterwards, to the contagion of the Small-pox in every way they could devise, with perfect safety from its infection, and that they have never been able to trace any subsequent diseases, as excited by Vaccination in the constitution.

"They feel it the more incumbent upon them, at this time, to make so public an avowal of their sentiments, as the Small-pox has lately been extremely prevalent, and fatal in this town and its vicinity, and because the prejudices against vaccination, particularly amongst the lower orders of the people, have been kept up by false and infamous reports, some of which have been artfully introduced into publications of notoriety and extensive circulation.

"They are fully convinced, that it is only by the unbiassed declarations of experienced and respectable practitioners in different districts, the public mind can be satisfied, and this most valuable discovery and practice universally adopted.

"Resolved, That this Society views with abhorrence the subtle, artful, and designing conduct of some practitioners, who continue indiscriminately to recommend Inoculation for the Small-pox or Cow-pox, as best suits their purposes, and by that means frequently, when the former is adopted, diffuse and spread that most dreadful and destructive pestilence through whole villages or populous districts, regardless what misery and distress they entail upon others; of such shocking conduct the Members of this Society have lately had too many proofs, and they earnestly hope that proper and efficient steps will ere long be taken by the Legislature to stop this horrid traffic.

*Salus populi suprema lex.*

"Resolved

"Resolved, That Copies of these Resolutions be transmitted to the different Medical Journals now in circulation, and a sufficient number of them printed and circulated in this town and its vicinity.

S. R. Newell, Colchester.

Ph. Gretton, ditto.

John Godfrey, Coggeshall.

T. C. Harrold, Nayland.

Henry Nunn, Maningtree.

Wm. Travis, East Bergholt.

G. Rogers, Manningtree.

Wm. Silke, ditto.

Benj. Smith, Wivenhoe.

Nath. Salter, Boxford.

Maurice Mason, St. Osyth.

R. Nunn, Colchester.

F. Eagle, Coggeshall.

G. Kemball, Tolleshunt Darcey.

T. Osmond, Thorpe.

*Copy of a Letter from* P. de Bruyn, Esq. *to* Thomas Wheeler, Esq. *dated November* 4, 1808.

"Reading an account in the Medical and Physical Journal of this month, that you have been engaged in examining some poisonous fungi, I beg leave to send you a specimen of what appears to me to be the agaricus glutinosus, for the purpose of comparing it with those fungi which lately proved so deliterious at Mitcham. Mine, when fresh, on being bruised, became very gluish, and it differs both in colour and figure from those 'poisonous fungi' delineated in the above Journal.

"An instance occurred two years ago, where a man and his wife, after supping on these supposed champignons, gathered in Hyde-park, were almost immediately attacked with giddiness, head-ache, dilated pupils, and redness of the conjunctivæ. The woman, who had eaten most, had the additional symptoms of being slightly insensible, and of a stretching out of the arms and legs, so that it was not without difficulty she could be made to sit down. These were the leading symptoms. Within a quarter of an hour I saw them, and gave repeated doses of vitriolated zink and antimonium tartarizatum, before a satisfactory vomiting could be procured. Afterwards purgatives were taken, and by the following morning they quite recovered."

On the above useful communication, we have Mr. Wheeler's authority to remark, that though the poisonous fungus is considered by some as a variety of agaricus glutinosus, yet the difference is too permanent to be admitted as a mere variety. The pyramidal cacumen and the tortuous stem are the striking marks of the poisonous, which has hitherto had no discriminating name, unless it is the *agaricus cacumenatus* of Withering, of which, unfortunately, no figure is given.

The *agaricus glutinosus*, besides being without the cacumen, is covered with a glutinous substance, which shows itself without being bruised. The poisonous fungus was found without any viscidity on its surface. The specimen sent by Mr. de Bruyn is the true poisonous fungus, and the supposed difference remarked by that gentleman, shows the extreme difficulty of delineating living objects, objects, in all of which, even of the same species, there are shades of difference.

---

The following paragraph, copied from the Journal de l' Empire of the 7th instant, might lead us to believe that dangerous mushrooms have been more frequent this season than usual in other parts of Europe. It is much to be regretted that no description is annexed of the fatal production,

*Pithiviers, 5 Novembre.*

Un habitant de la commune de Boynes, canton de Pithiviers, département du Loiret, étant allé dans un bois voisin pour y ramasser du bois sec, mena avec lui ses deux filles, âgees de 10 à 12 ans. Ces enfans trouvèrent une si grande quantité de champignons, et si beaux en apparence, qu'ils ne purent résister au desir d'en emporter une ample provision. Le soir, la mère apprêta les champignons, dont toute la famille mangea avec abondance, excepté le père, qui ne fit qu'y goûter, par complaisance, aimant mieux, dit-il, manger de la viande.

Vers deux heures après minuit, de violentes coliques, accompagnées de convulsions, se manifestèrent chez la plus jeune des filles, qui mourut après six heures de déchiremens d'intestins. Le même jour, la seconde la suivit : après les mêmes symptômes, on appelle le curé du lieu, qui se transporte au sussitôt. Son premier soin est d'interroger le père et la mère, qui, ne se sentant point dans leur état ordinaire, étoient restés au lit. Après plusieurs questions réitérées, ils avouérent enfin qu'ils avoient mangé des champignons la veille. Alors, on manda un chirurgien, qui ordonna sur-le-champ les remèdes convenables; mais ils furent administiés sans aucun succés. Les malades périrent le lendemain. Il est à remarquer qu'un enfant à la mamelle, qui avoit été allaité dans la première nuit, fut aussi victime des suites du poison.

---

Dr. Adams has received a sample of opium from Madeira, the produce of Porto Santo, one of that cluster of islands. It is said to be the natural juice of a poppy yielded by incision. Its smell is much more aromatic than in the common opium. It is also free from every impurity. Trial has been made with it on a cancerous patient, who, from the long habit of using opium, was thought a fit subject for comparison. The following is the account given by the surgeon who attends her.

" Mr. ——— presents his compliments to Dr. Adams, and immediately upon receiving his letter, furnished their patient Mrs. ——— with a box of pills made with the opium he was so good as to send him. The first night she took them, she experienced an uncommon good night ; but since that he has given her the Madeira opium and the common alternately, and she has not been able to discover the least possible difference, though she has been very accurate in her accounts.

*Clapham, Wednesday Night.* Mr.

Mr. William Skrimshire, jun. has made some observations on the fecula of potatoes, and some other British vegetables, which, during the present high price of bread, seems particularly worthy of attention. One thousand grains of the former roots yielded 111 grains of fine white fecula, when perfectly dry, which he recommends not only as the most economical means of fattening cattle and pigs, but also as a very palatable and nutritious food for man. This fecula, which is generally known to laundresses by the name of potatoe starch, is obtained by the process which they employ. Formed into small cakes, and dried in the open air, or by a gentle heat, this preparation will keep for many years. When the fecula and pulp are mixed together, and thus prepared, half an ounce of it will, says Mr. Skrimshire, gelatinize so large a quantity of boiling water as to afford a sufficient meal for any labouring person in health. It may be sweetened either with molasses or sugar; or being boiled with an onion or pot-herbs, and seasoned with pepper and salt, it will make a very palatable, wholesome, and nutritious soup. If this preparation be boiled with milk, sweetened with sugar, and flavoured with a little wine or spice, it forms the most nourishing and restorative food that can possibly be administered to the sick and convalescent. From the ease with which it is digested, it is peculiarly adapted to the impaired organs of the debauchee, and the feeble powers of infancy. With a larger proportion of the preparation, a stiff jelly may be formed, which acidulated with lemon-juice, or any other vegetable acid, becomes the best domestic remedy that can be employed in every species of sore throat. The pure fecula, the author asserts, will be found superior in every respect to salep, sago, arrow-root, or any of the vegetable preparations of that kind, which have been so pompously advertised and recommended to the public by persons interested in the sale of them. Another use to which Mr. Skrimshire has applied potatoes, is likewise worthy of notice: "I have frequently formed a very grateful and nutritious beverage (says he) from potatoes sliced, roasted to a coffee colour, then ground in a mill, and mixed with a sixteenth of its weight of the best Turkey coffee." The other vegetable productions on which Mr. Skrimshire has made experiments, are the horse-chesnut, acorns, and the root of the red-berried briony, commonly called mandrake, and of the cuckow-pint, or walk-robin. All of these yield a large proportion of fecula, which forms a nutritious food for man or other animals.

---

The French chemists have not only repeated Mr. Davy's experiments on the decomposition of alkalis, but have confirmed the accuracy of his researches, by obtaining similar results by a different process. Messrs. Gay and Thenard have succeeded in deoxidating potash by means of iron. The event is announced in *Correspondence sur l'Ecole Imperiale Polytechnique*, Number 10, in the following terms: "A letter from London, dated November 23, 1807, announced that Mr. Davy had succeeded, by means of a strong galvanic pile, of decomposing the two alkalis of potash and soda; and and that he had read a memoir to the Royal Society, in which he concluded that these two alkalies were metallic oxides. On the 8th of December, Messrs. Gay and Thenard, repeated Mr. Davy's experiments at the laboratory of the Polytechnic School, and actually obtained, at the negative pole of a pile with large plates, the two new metals, the existence of which had not even been suspected previous to Mr. Davy's experiments. The above chemists, however, continued the inquiry in a new point of view. They proposed to themselves the discovery of a substance sufficiently oxidizable to take off the oxygen from the alkalies, which had been ascertained to be metallic oxydes, and their experiments were attended with the greatest success. On the 7th of March, 1808, they informed the Institute of France that, upon treating potash with iron, in the fire of a reverberating furnace, the iron deoxidated the potash, and made it pass to the metallic state.

---

An experienced propagator of trees, shrubs, and plants, has discovered a cheap and efficacious method of propagating, by cuttings, all kinds of fruit trees, without the aid of artificial heat. By this novel and advantageous system, it appears that we are not only enabled with certainty to propagate any particular species, but preserve, with the strictest purity, the more valuable fruits, without liability to adulteration or degeneracy, the certain consequence of budding or grafting upon ungenial and improper stocks, and avoid the common inconvenience of receiving erroneous sorts from public nurseries.

---

Dr. Clough, 68, Berner-street, will, on Monday the 9th of January, 1809, at ten o'clock in the morning, resume his Course of Lectures, on Puerperal Anatomy, Physiology, and Pathology. —A Syllabus and Prospectus of which, may be had of Dr. Clough, as above; J. Callow, Crown Court, Soho; and at the St. Mary-le-bone General Dispensary, No. 77, Welbeck-street, Cavendish-square.

An Evening Course for the convenience of such Students as may be otherwise engaged in anatomical pursuits, will be given on Tuesdays, Thursdays, and Saturdays, at Eight. The first Evening Lecture commencing on the 10th of January 1809.

---

Dr. Lambe has in the press, Reports on the Effects of a peculiar Regimen on Cancerous Tumours and Ulcers. These Reports will appear early in the ensuing month.

Mr. Charles Sylvester, of Derby (late of Sheffield), has in the press an Elementary Treatise on Chemistry, the plan of which is in many respects original.

Mr. Taunton, Surgeon to the City and Finsbury Dispensaries, is about to publish a small work on Pathology, which will be illustrated with Engravings.

